# Supplementary material for: RNase III cleavage sites spread across splice junctions enforce sequential snoRNA processing
Source: EMBO Rep. 2025 Aug 26;26(19):4675–90. doi: 10.1038/s44319-025-00553-y (PMC12508059; doi:10.1038/s44319-025-00553-y)

Figure 1.B: uncropped Northern blots of Pac1-deficient strains (replicate #1)

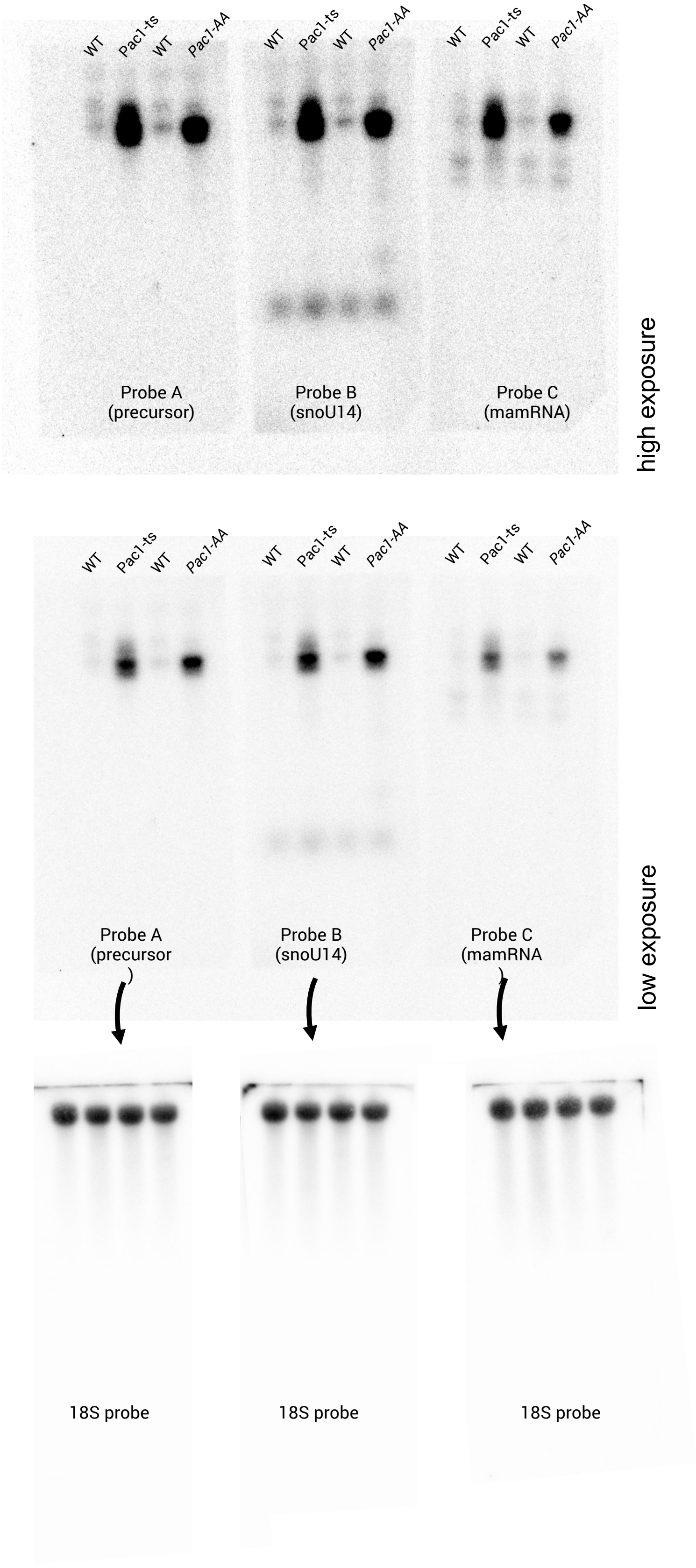

Figure 1B: uncropped Northern blots of Pac1-deficient strains (replicate #2, shown in figure EV1B)

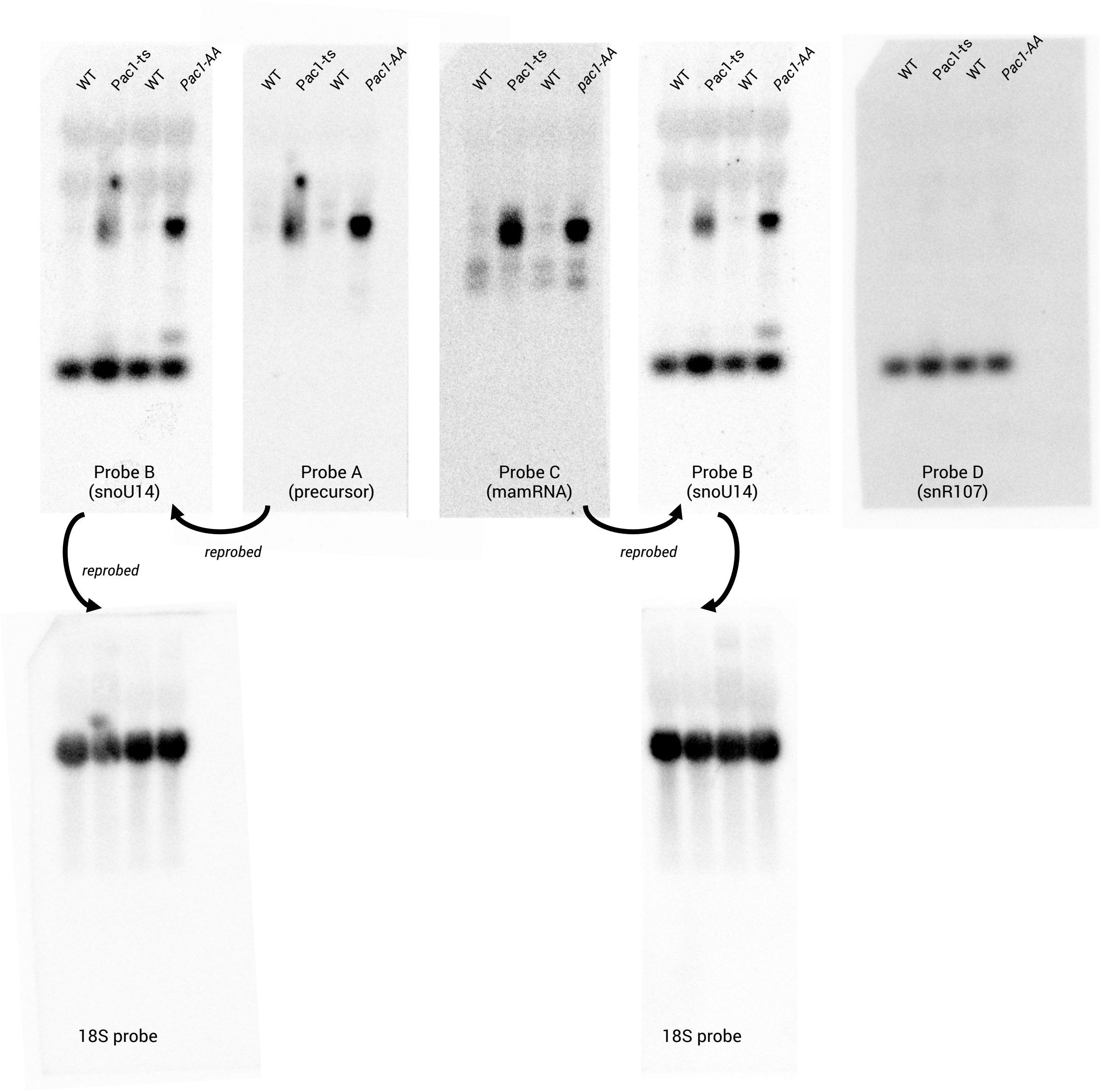

Figure 1B: uncropped Northern blots (additional replicates #3-4-5, not shown in main figures)

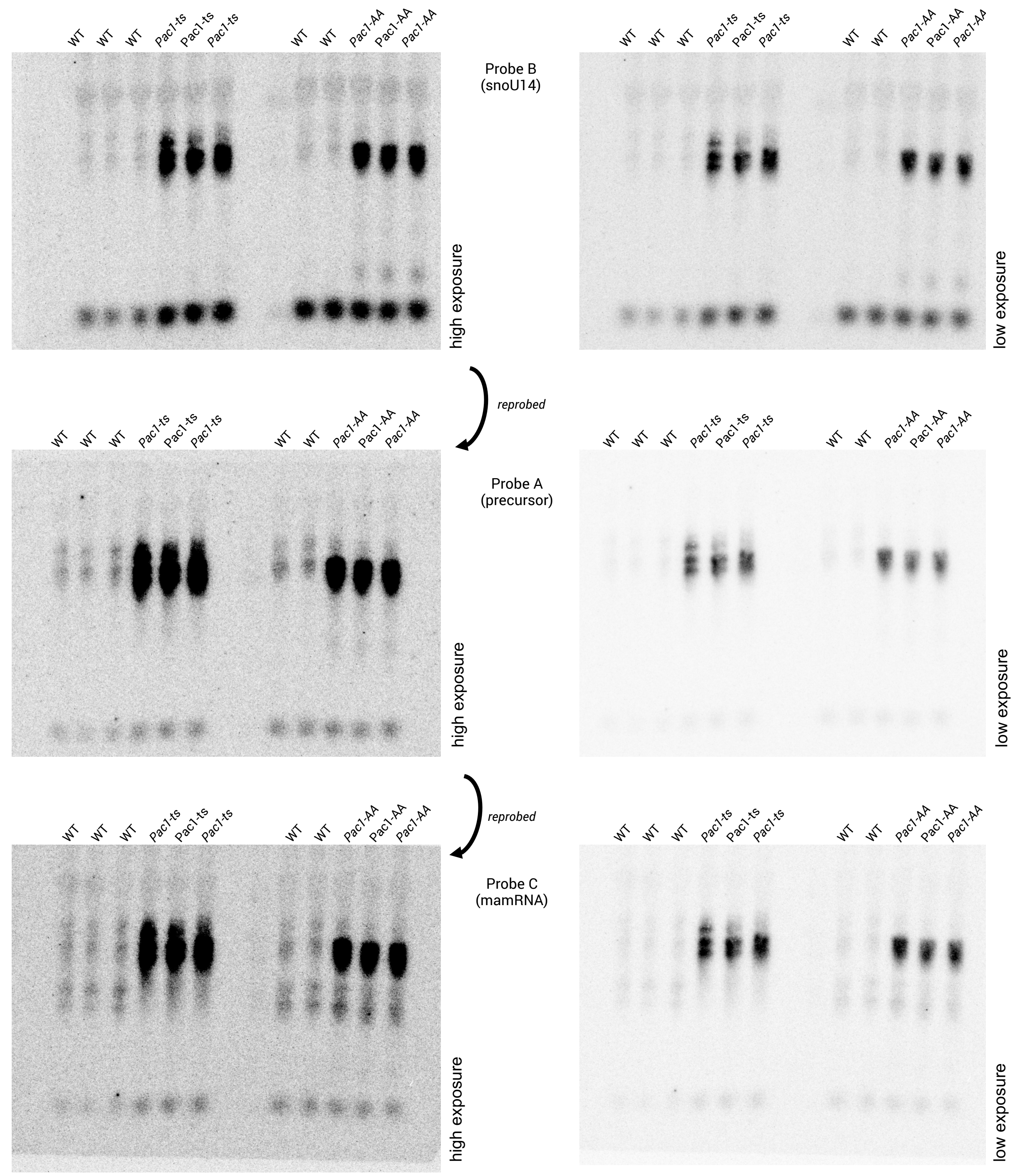

Supplement: Supplementary file 5 — Source data Fig. 1 [file 44319_2025_553_MOESM5_ESM.zip › Figure 1/1B/uncropped_blots_figure1B.pdf]
