# Supplementary material for: RNase III cleavage sites spread across splice junctions enforce sequential snoRNA processing
Source: EMBO Rep. 2025 Aug 26;26(19):4675–90. doi: 10.1038/s44319-025-00553-y (PMC12508059; doi:10.1038/s44319-025-00553-y)

Figure 2.B: uncropped Northern blots of *StemDead* strains (replicate #1)

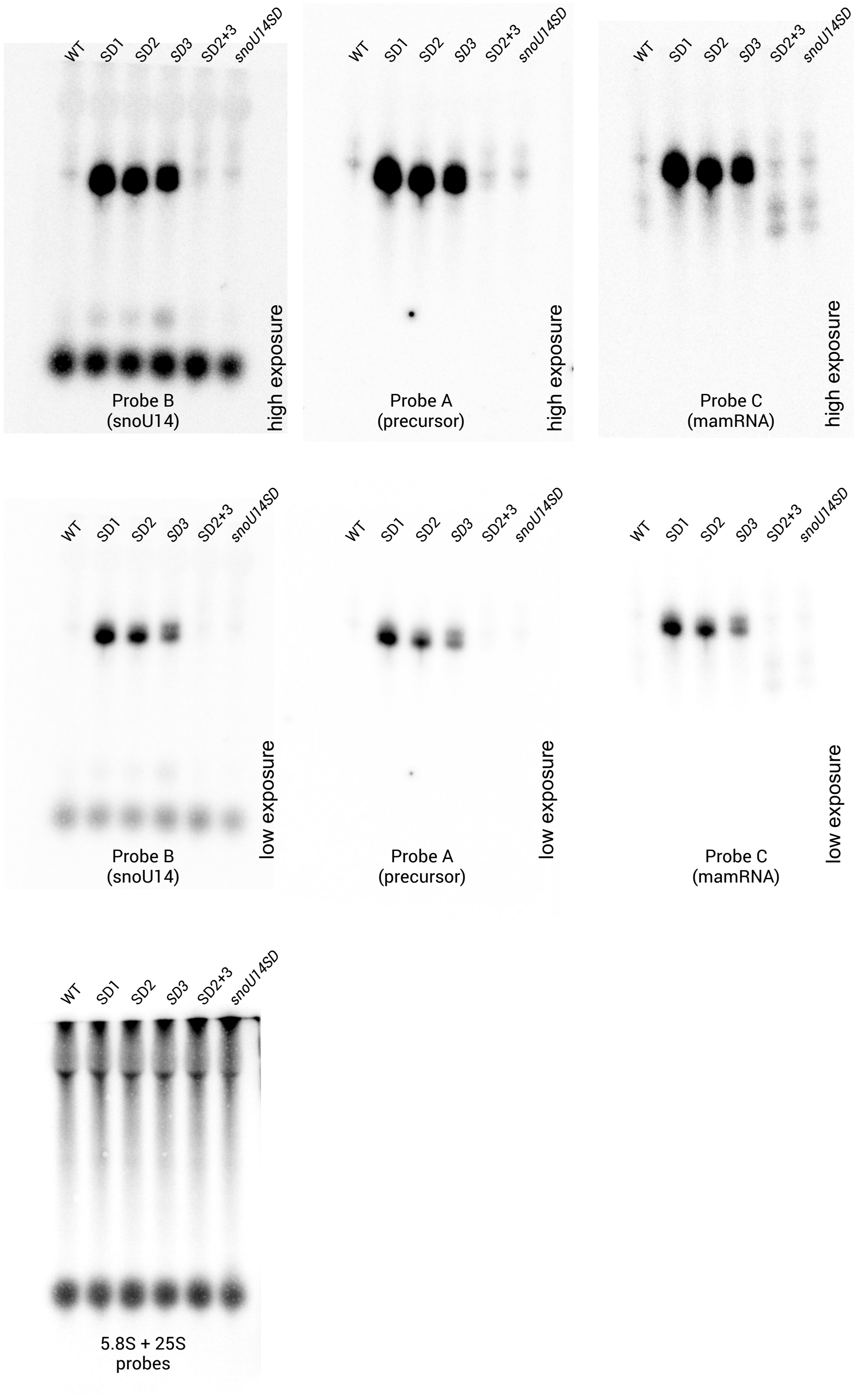

SOURCE DATA, related to... Migeot et al., 2025

Figure 2.B: uncropped Northern blots of *StemDead* strains (replicate #2, not shown in main figure)

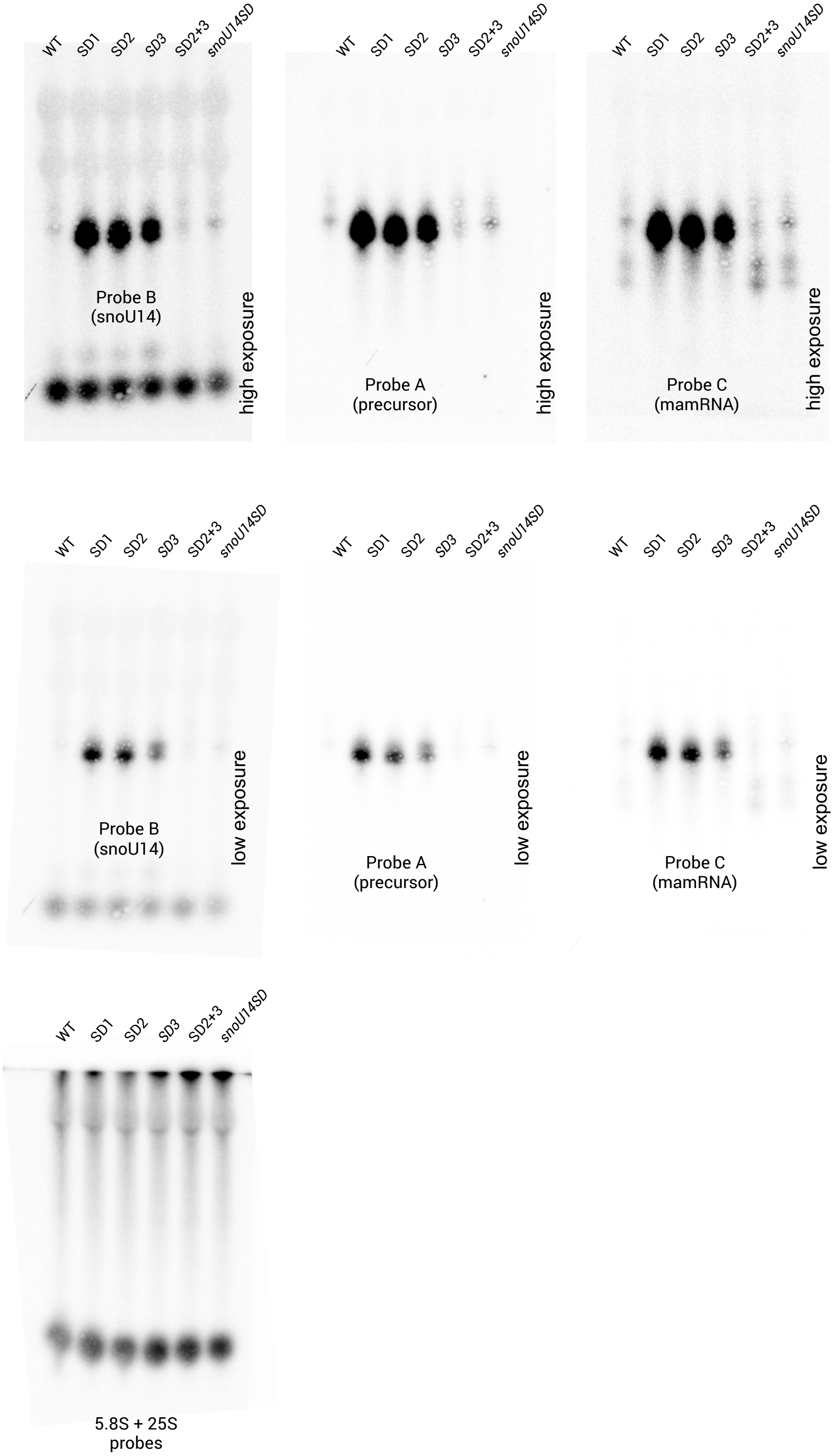

Supplement: Supplementary file 6 — Source data Fig. 2 [file 44319_2025_553_MOESM6_ESM.zip › Figure 2/2B/uncropped_blots_fig2B.pdf]
