# Supplementary figures and images for: RNase III cleavage sites spread across splice junctions enforce sequential snoRNA processing
Source: EMBO Rep. 2025 Aug 26;26(19):4675–90. doi: 10.1038/s44319-025-00553-y (PMC12508059; doi:10.1038/s44319-025-00553-y)

Figure 3B: uncropped Northern blots of *ConStem* strains (replicates #1 & 2)

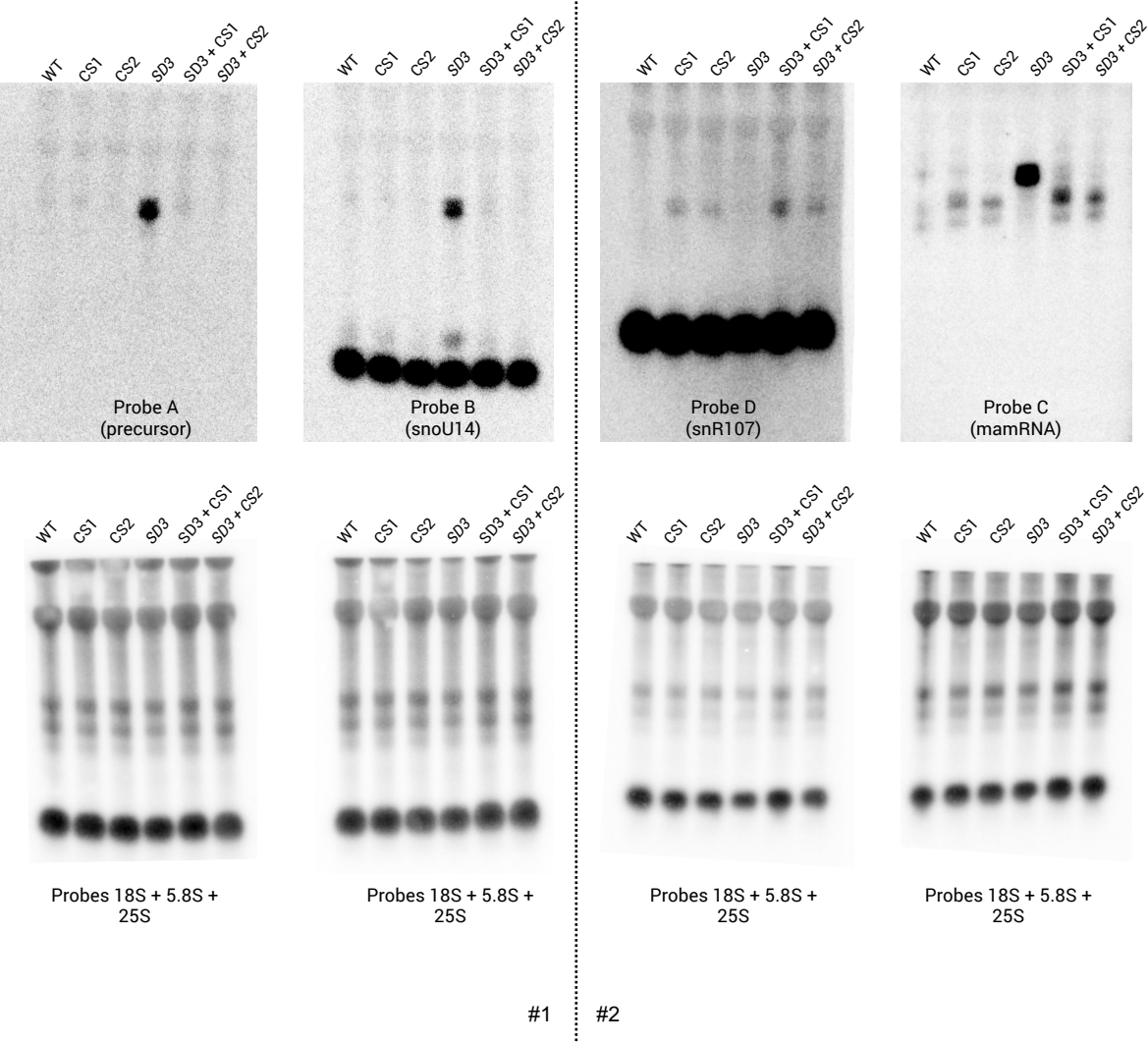

Supplement: Supplementary file 7 — Source data Fig. 3 [file 44319_2025_553_MOESM7_ESM.zip › Figure 3/3B/uncropped_blots_fig3B.pdf]

Figure EV3A: uncropped rRNA Northern blots of *StemDead* strains (replicates #1 & 2)

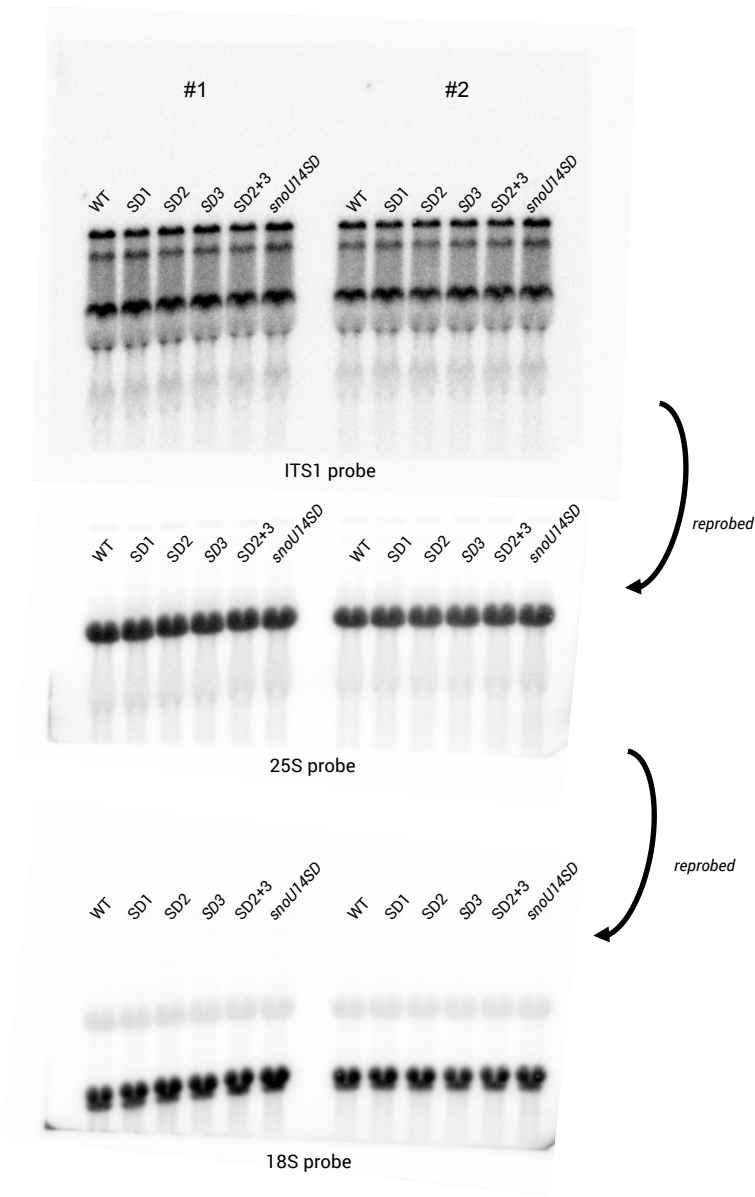

Supplement: Supplementary file 8 — Figure EV3 Source Data [file 44319_2025_553_MOESM8_ESM.zip › FigureEV3/EV3A/uncropped_blots_figEV3.pdf]
