# Supplementary material for: RNase III cleavage sites spread across splice junctions enforce sequential snoRNA processing
Source: EMBO Rep. 2025 Aug 26;26(19):4675–90. doi: 10.1038/s44319-025-00553-y (PMC12508059; doi:10.1038/s44319-025-00553-y)
Supplement: Supplementary file 9 — Expanded View Figures [file 44319_2025_553_MOESM9_ESM.pdf]

Expanded View Figures

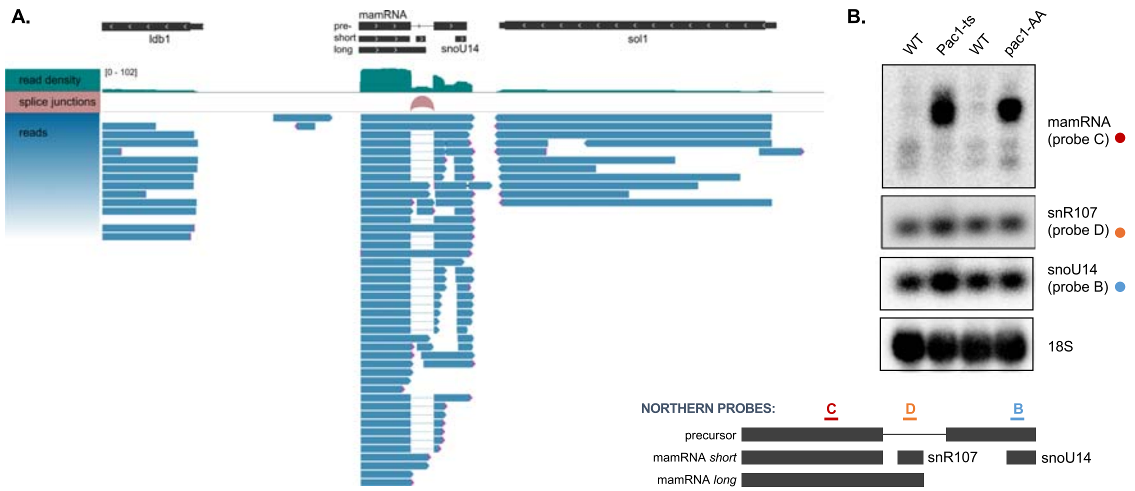

Figure EV1. A common precursor for mamRNA and snoU14 depends on Pac1 activity.

(**A**) Read density, splice junctions and individual reads from a long read sequencing experiment (wild-type strain) over the mamRNA/snoU14 locus. (**B**) Representative northern blot analysis of transcripts originating from the mamRNA/snoU14 locus in conditions where the activity of Pac1 is compromised. Probes positions are indicated below (**B**).

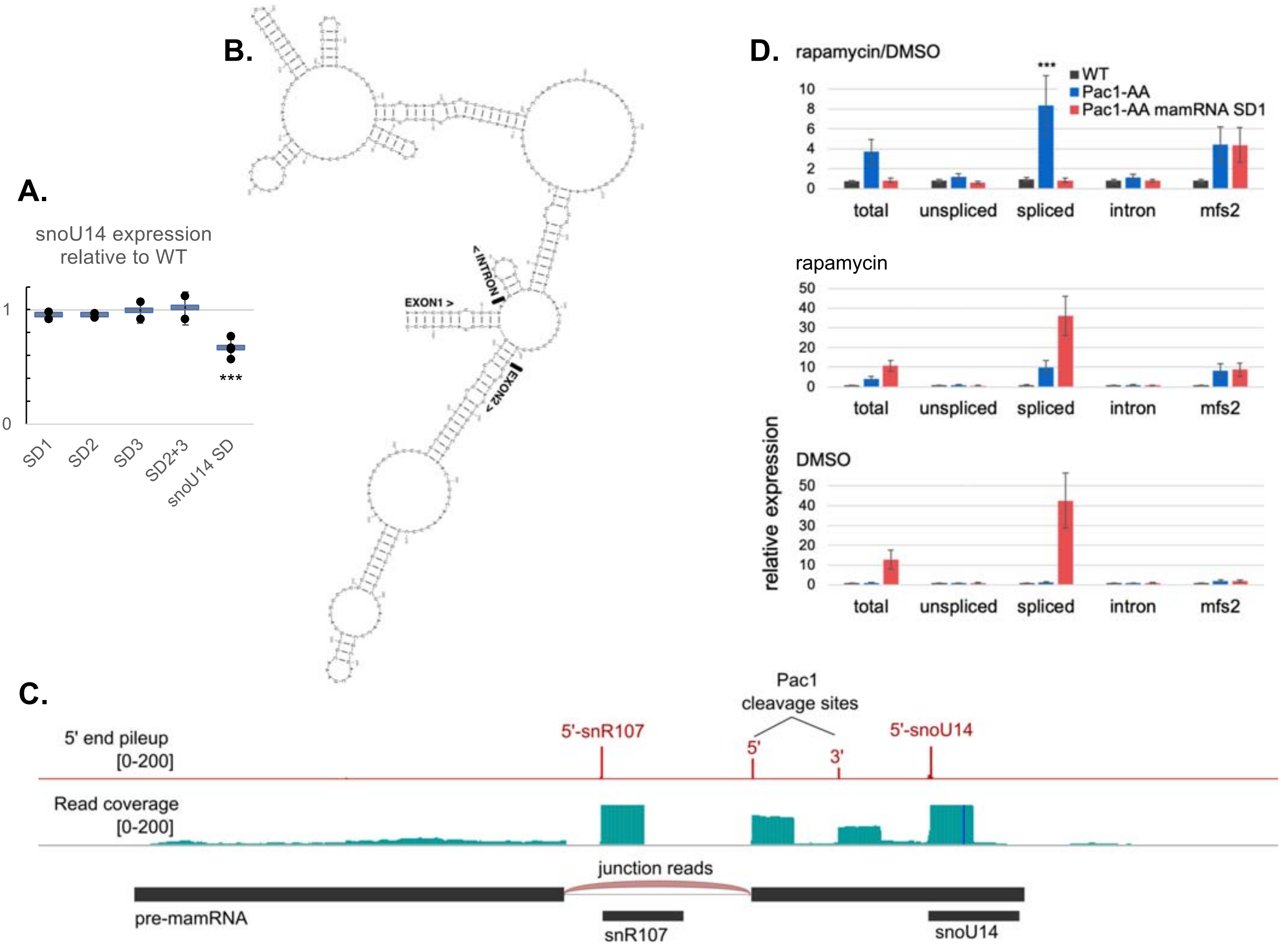

Figure EV2. A stemloop spanning the exon–exon junction directs Pac1 cleavage at the mamRNA.

(**A**) Semi-quantitative determination of mature snoU14 relative expression from pixel measurements of the northern blot experiments presented in Fig. 2B. Individual replicates ($n = 4$) are represented by filled circles. Means +/− SD are depicted by gray rectangle with error bars. Statistical significance of the differences in mature snoU14 level in the snoU14-SD mutant compared to the WT strains is indicated (***: Student's t test P value = 0.00046). (**B**) Predicted secondary structures of the unspliced pre-mamRNA/snoU14 transcript. (**C**) 5′-end pile-up of reads and overall coverage from a degradome-seq experiment in wild-type strain (SRR12004691 (Zhang and Pelechano, 2021)) over the mamRNA-snoU14 locus. The putative 5′ and 3′ Pac1 cleavage sites are indicated. (**D**) RT-qPCR analysis ($n = 3$) of transcript isoforms spanning the mamRNA locus in the indicated strains treated for 2 h with rapamycin (middle panel) or with its solvent (DMSO) as control (bottom panel). The qPCR amplicons are the same as in Fig. 1D with the addition of mfs2 as a positive control - mfs2 have been previously shown to be upregulated by conditional nuclear exclusion of Pac1 by rapamycin in the Pac1-AA strain (Yague-Sanz et al, 2021). The fold change and statistical significance of the rapamycin treatment effect compared to the DMSO control are indicated for the spliced pre-mamRNA isoform (top panel, ***: Student's t test P value = 0.00031). Error bars represent the standard deviation of the mean.

 © The Author(s)

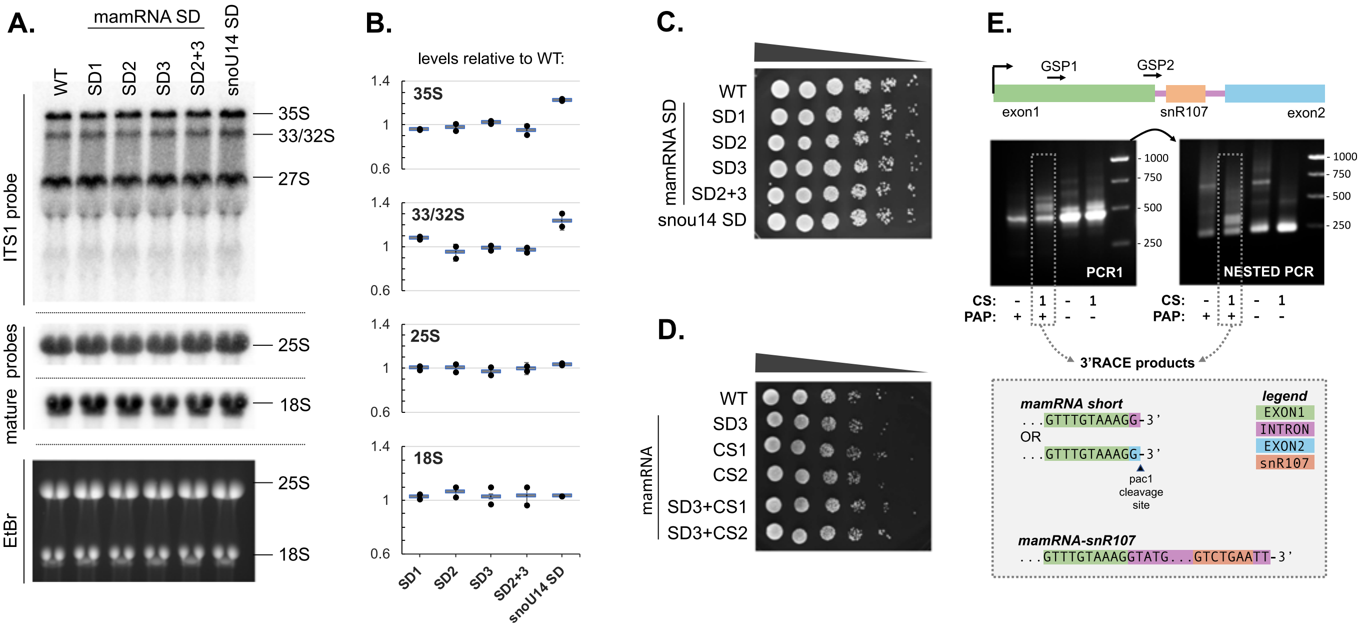

Figure EV3. snoU14 matured independently from Pac1 is functional.

(**A**) Northern blot analysis of pre-rRNA transcripts in the indicated strains with internal transcribed spacer (ITS) probe. Mature rRNA forms (25S and 18S) are revealed by ethidium bromide (EthBr) staining. (**B**) Semi-quantitative determination of mature 35 and 33/32S rRNA precursor relative expression from pixel measurement of the northern blot experiments presented in (**A**). Individual replicates are represented by filled circles ($n = 2$). Their mean is depicted by a gray rectangle. (**C**) 5-fold dilutions of yeast cultures from the indicated strains spotted on YES-agar plates and incubated for 2 days at 32 °C. (**D**) 10-fold dilutions of yeast cultures from the indicated strains spotted on YES-agar plates and incubated for 3 days at 32 °C. (**E**) 3′-RACE analysis of mamRNA isoforms from total RNA extracted from CS1 mutant (CS) treated with poly(A) polymerase (PAP). Gene-specific primer (GSP) 1 and 2 were used in PCR1 and nested PCR, respectively. In this experiment, the mamRNA short isoforms ended one nucleotide shorter than currently annotated, with a G that could correspond either to the first nucleotide of the intron, or to the first nucleotide of exon2, in which case the 3′-end of the isoform matched the predicted 5′ Pac1 cleavage site. Source data are available online for this figure.

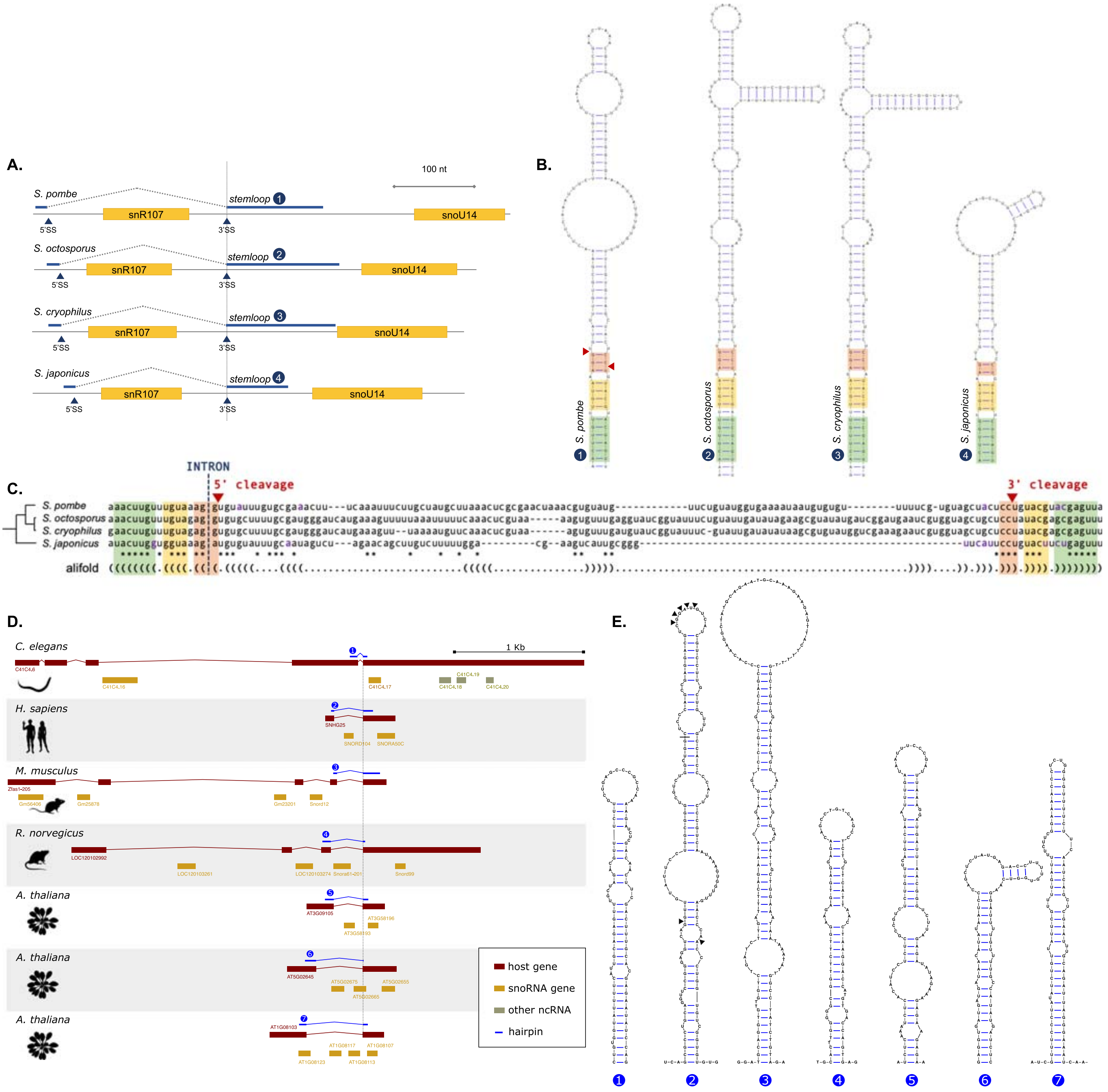

Figure EV4. Conservation of secondary structures spanning splice junctions in mixed snoRNA clusters.

(**A**) Genomic arrangements of snoU14 orthologs in *Schizosaccharomyces* species, aligned to their upstream 3′ splice site (3′SS). Unannotated snR107 orthologs were positioned in the upstream introns based on the conservation of C and D boxes, but with the exception of *Sp*snR107, their exact boundaries are uncertain. (**B**) Predicted secondary structure of the sequences spanning the exon-exon junction in (**A**). Arrowheads indicate the Pac1 cleavage site identified in *S. pombe*. Colored boxes indicate particularly conserved regions, as shown in (**C**). (**C**) Multiple sequence alignment of the predicted stem loops shown in (**B**). Conserved nucleotides are indicated by a star (*) and the consensus secondary structure of the alignment (alifold) is represented in dot-bracket notation. Red arrowheads indicate the Pac1 cleavage site identified in *S. pombe*. The exon-exon junction is indicated by a dotted blue line. Colored boxes indicate particularly conserved regions. Nucleotides that diverge from the consensus sequence while preserving the conserved structure are colored in purple. (**D**) Genomic arrangements of selected genes host to both intronic and non-intronic snoRNAs, aligned to the 5′ end of their last exon. (**E**) Predicted secondary structure from the sequences spanning the last exon–exon junction of host genes displayed in (**B**). Cleavage sites on SNHG25 inferred by degradome-seq experiments (stemloop #2) are indicated by arrowheads.

 © The Author(s)
